# Notes on Gynæcology and Gynæcologists Abroad

**Published:** 1878-04

**Authors:** 


*Mr. Editor:*—I notice in the January number of the JOURNAL AND EXAMINER a case of ovariotomy reported by Dr. E. R. Willard, of this city, as having occurred in *his* private practice. Now I wish to make some corrections in regard to the case. In the first place, the case belonged to me, and has been under my care since April 25, 1875, to this date, and Dr. Willard did *not see* or *treat* the case from that date until about October 20, 1877, when I invited him to visit the case with me and examine it with reference to an operation. The lady visited Dr. A. W. Heise with me on May 26, 1876, who advised her, at that time, to let it alone. About August 20, 1877, she again visited Dr. Heise, who advised paracentesis, which was performed by me as stated by Dr. W., on August 28, and September 30, 1877. On October 26, 1877, I performed the operation of ovariotomy, *assisted* by Dr. E. R. Willard and his son G. E. Willard. Dr. W. seems to have been in great haste to report the case, and whether intentionally or not, to claim all the honors. I desire to call the attention of the readers of his statement to a full report of the case, which will soon appear in the *Philadelphia Medical and Surgical Reporter*.

D. J. MERRIMAN, M. D.

WILMINGTON, ILL., Feb. 13, 1878.

[We are pleased to furnish the opportunity of correcting the apparent error, to which attention is directed in the above communication, and must adhere to our rule by declining to print any further communication on the same subject, in order to avoid a controversy in which the mass of our readers are not interested. —ED.]

### NOTES ON GYNÆCOLOGY AND GYNÆCOLOGISTS ABROAD.

(*From our Foreign Correspondent.*)

VIENNA, February 26, 1878.

After more than twenty years spent in active practice, I have come abroad with, I hope, few deeply rooted prejudices of my own, and with a desire to impartially observe the treatment of disease by the best men in Europe, in the hope of receiving benefit from what I shall see and hear.

On account of the many conflicting opinions which I have found entertained by these great lights in our profession, and more especially in that branch of our art which I have named as the title of my letter, I have more than once envied that man, who being deeply grounded in some particular medical faith, hobby or theory, is able to look down with an eye of profound commiseration on all who are outside of the pale of his contracted mental horizon. These conflicting views pertain not only to the treatment, but to the nature and pathology of uterine diseases.

One gynæcologist, for example, exhibits a case of chronic corporeal metritis, the length of the body of the womb and the character of the discharge, all combining to render such a diagnosis as clear to him as the sunlight in heaven.

Another decides that this identical pathological condition is due to endometritis, stoutly maintaining that chronic metritis proper, is as rare an affection according to his observations, as elephantiasis in Lapland.

Still another calls attention as he looks through the speculum, to a typical case of ulceration of the cervix, which his neighbor calls a simple erosion and so on, ad infinitum.

In treatment the best authorities are no less at variance. One uses with impunity, instruments and medication, that another not only disapproves of, but considers dangerous to life.

The first gynæcologist of note whom I interviewed after landing in Europe, was Dr. Athill, of Dublin, whom it is unnecessary for me to introduce to the intelligent reader of this journal.

After the simple presentation of my card, he received me with that warm hospitality so characteristic of the cultivated Irishman.

He is a little spare built, dark complexioned man, apparently about forty-five years of age, and as full of energy and life as Barnum in his palmiest days.

His local treatment of what Dr. Thomas calls glandular erosion of the cervix, is an application of two parts of carbolic acid and one part of alcohol.

He makes these applications once or twice a week, irrigating the parts twice a day freely with warm or tepid water.

This process of irrigation of the mouth of the womb, is rendered more efficient, as well as greatly simplified, by using the bed pan which Dr. Athill has constructed, and which is illustrated in the last edition of his very excellent little work on uterine diseases.

He is very emphatic in his condemnation of the use *locally*, of the preparations of iron and other powerful astringents to the womb, on account, as he thinks, of their liability to obstruct the circulation in the parts, producing thrombi, and the secondary train of evil consequences that are likely to follow. Showing me a case that was typical of what many American gynæcologists recognize as a laceration of the muscular fibers of the cervix, and treat by paring the edges of the flaps and bringing them together with sutures, I asked Dr. Athill what he thought of the procedure, and if he had ever tried it. His reply was, that although American physicians had done much to advance gynæcology, that just now, following in the wake of Dr. Sims, we were a little crazy on this subject of uterine surgery, and when a case presented itself we lost sight of every therapeutical appliance except the knife.

The position that Dr. Athill holds as master of the Rotunda is one of the best in Great Britain. The fund left to pay the master, by the founder of this great charity, now amounts, I believe, to $40,000 per year.

The gynæcological department is a new feature in this institution, but one that is being rapidly enlarged through the energy of Dr. Athill.

Professor Alexander Simpson, of Edinburgh, is a nephew of the world-renowned Sir James Simpson, and is a good example of what energy and hard work will develop, even where original talent was not perhaps of the highest order.

He came to this city as a poor boy, studied medicine with his uncle, and now occupies the position that Sir James had hoped to hand down to one of his own sons—but instead of that, one of them is an attorney with few clients, and the other is a spendthrift, who lives mostly on the Continent, and leads a life, if report be true, not calculated to shed lustre upon the name he has inherited.

Prof. Simpson did not strike me as a man who would ever extend our knowledge of gynæcology by original investigation; he seems to be content to follow in the footsteps of his distinguished uncle. He is a skillful operator, very tender and humane toward his patients, and greatly esteemed in society for his many worthy traits of character.

Prof. Matthews Duncan is in many respects the opposite of Prof. Simpson. He is rough and austere in his treatment of his hospital patients, and there seems to be but little cordiality between him and his medical class. He seemed inclined to attribute most uterine diseases to pathological changes in the os and cervix, and to treat them mainly by the application of pure nitrate of silver. Seeing many cases of uterine fibroid tumors in his wards, I asked the doctor what had been his experience in the use of ergotine hypodermically in the treatment of this affection. He said that he had tried it but it had done no good at all. I have been much interested on this point, and have repeated the same question to all the promenent men whom I have met and find none of them have any faith in the remedy. Prof. Simpson is the only man whom I saw using it.

Prof. Duncan is the most thorough skeptic as to the therapeutic value of drugs of any physician I have met. In his wards were a number of cases of well-marked chlorosis. Noticing that no medicine was given them, I asked, "Professor, do you not use iron in chlorosis?" His reply was, "*I never give iron*; it is one of the delusions of medicine. We laugh at the infinitesimal dogmas of homeopathy, and yet nine hundred and ninety-nine out of every thousand of old-school physicians go on from year to year giving iron on a purely chemical theory, with not a whit of evidence in support of its virtues as a remedial agent."

He keeps his patients suffering from this disease quietly in bed, feeds them well, has the wards well ventilated, but ignores completely the value of medicines. During the last summer Prof. Duncan has been appointed to a professorship at St. Bartholomew's, in London, and as he and his friends have always felt that young Simpson's appointment to the chief professorship in the University of Edinburgh, was an injustice to him, his removal from here will greatly add to the harmony of this faculty.

Prof. Barnes, of London, is a typical "John Bull," large in frame, with a red face and nose that would seem to indicate free indulgence on the part of its possessor in a generous diet of beef and beer. For chronic endo-metritis his favorite local treatment is the tincture of iodine. If there coexists an involvement of the cervix, he applies to this part the nitrate of silver; but if either of the above diseased conditions is complicated with any marked displacement of the womb, he usually applies a pessary with a view to remedying this before instituting any local treatment. He usually employs some modification of the Hodge pessary, applying it to his charity patients who have often to walk a long distance to their homes; a procedure which he claims to be perfectly safe if you are only sure that your instrument is accurately fitted.

M. Guérin, of Paris, the great cotton man in surgery, has utilized this agent in the management of his gynæcological patients. He treats his cases of chronic ulceration and erosion of the os uteri, by cotton pessaries made in a conical form with a depression in the smaller end, which he fills with an astringent ointment of either tannin or one of the salts of iron. He applies this directly to the parts through a speculum, keeps his patient quietly in bed for some weeks, and his results, so far as a temporary healing of the parts is concerned, are very good indeed. The pessaries are changed once or twice in the twenty-four hours, depending upon the amount and character of the discharge.

I asked the doctor if these patients were not subject to relapses after resuming their ordinary avocations.

He answered that they would not if they remained under treatment long enough, that is from one to two months.

M. Guérin uses thick layers of cotton batting for all surgical dressings, to fulfill the same indications for which Mr. Lister employs his antiseptic gauze; and I will so far digress from my subject as to urge upon the reader, the adoption of his mode of treating severe burns.

The plan pursued is to apply freely to the burned surface a mixture of olive oil and lime water, and then envelop the parts in a thick layer of cotton batting, over which is applied a well fitting bandage. The first dressing is left on for from twenty-four to forty-eight hours, and then re-applied. After from six to ten days, the lime water and olive oil dressing is changed, and in its stead is used either an oxide of zinc or mild astringent ointment, according to the amount of the discharge and the condition of the wound.

The batting should be of the best quality, and the layer applied should be from 6 to 8 inches thick before the bandage is put on.

M. Guérin claims that by totally excluding the atmospheric air during the process of healing, the development of cicatricial tissue, which often proves such a source of evil in extensive burns, is greatly diminished.

I have seen patients brought into the Hôtel Dieu with extensive burns, suffering the most intense agony, in whose cases the relief from pain after applications of this dressing, was always immediate and permanent.

Professor Spaeth, of Vienna, is the best extemporaneous lecturer I have heard in this city.

His lectures are systematic in character, and include midwifery and the diseases immediately connected with the puerperal state.

Neither he nor Professor Karl Braun, ever allow any ergot to be given to a woman in child-bed before the expulsion of the fœtus, believing that the remedy acts toxicologically upon the unborn child, greatly endangering its life.

For puerperal convulsions he uses opium and chloral. Still, after reviewing the different modes of treatment that have been adopted in this century, he avers that statistics in Vienna show them all to have been equally efficacious, and as they had been so varied, he is more than suspicious that they were all equally *worthless.*

On January 25th, 1878, Professor Spaeth read a paper before the "Gesellschaft der Aerzte" of Vienna, on the subject of the Cæsarean section.

He said, in the course of his remarks, that in the numerous instances where this operation had been done in Vienna during the century, only two patients had recovered, one of whom he brought before the society, in whose case he had himself operated.

Professor Karl Braun's course is mainly clinical in character, and is attended mostly by physicians and advanced medical students.

He is a large indolent appearing man, weighing perhaps 250 lbs., and greatly resembling "Boss" Tweed. He sits in his easy chair, talks in a most deliberate, and often hesitating manner, and whenever he is at a loss for a word to complete a sentence, he says, "Nun meine Herren," which an inquisitive Yankee listener says he repeated eighty times in one hour. Professor Braun is a man of powerful intellect, undoubtedly standing at the head of the profession in his department in South Germany. Like Billroth of this city, and Langenbeck of Berlin, he operates with a boldness far exceeding anything I have ever seen in America.

The removal of the cervix uteri by the galvano-cautery, for malignant disease of the parts, is a procedure that Professor Billroth resorts to constantly. He claims that this operation has been very successful in his hands, and when early and efficiently performed, the disease is not likely to recur.

In operating he removes all diseased tissue, regardless of the integrity of surrounding parts.

There is a patient in the hospital now, apparently well, into whose peritoneal cavity he opened when removing a cancer of the cervix; and fistulous openings into the bladder after his operations, are not infrequent.

His mode of amputating the cervix, where there is simply an abnormal elongation of that part of the uterus, seems to me to be both simple and safe. He passes a straight needle armed with a double wire suture through the centre of the cervix, about one half inch above where he proposes to amputate. He then ties each one of these sutures firmly around each transfixed position of the cervix, so as to effectually cut off all circulation from the parts below. He next amputates the cervix with a scalpel, cutting half way through the parts from above downward, and the other half from below upward, the incisions being carried in each case, slightly from without inward, making a partial flap of mucous membrane to assist in covering the stump. The parts are then brought together by deep wire sutures, after which the first ligatures, passed in higher up to control the hemorrhage, are removed.

His cases, this winter, have all done excellently, and the bleeding has been insignificant.

I saw him operate on a woman, the third of three sisters who were sterile from an elongation of the cervix, and, as a similar procedure had remedied this condition in the first two, the third had applied to be treated in the same manner.

Professor Braun treats most of his cases of endometritis by first scraping out the entire intra-uterine surface with a sharp, spoon-shaped curette, and then injecting the womb with the liquor ferri chloridi.

The instrument that he uses, is a large hypodermic syringe, attached to the end of which is a hard rubber tube about the size of a No. 6 catheter, nine inches in length, slightly curved near its upper extremity, where it is perforated on the sides by a number of small apertures.

The quantity of the fluid used should vary from 15 to 30 m., according to the size of the uterine cavity.

The instrument is carried at first up to the fundus, and the fluid is slowly thrown in as it is being gradually withdrawn, so that by the time the fluid has all been injected from the syringe, the end of the instrument has nearly reached the external os.

The tube is now passed again slowly back to the fundus, and as this is being done, all fluid is again drawn into it, when the operation is completed.

His results in the treatment of some of his cases are very brilliant indeed.

To give a single example :

A woman about 30 years of age, unmarried and previously in good health, fell from a distance alighting upon her feet, and although no marked displacement of the uterus followed the accident, she complained ever aftewards of an uneasy and painful sensation about her pelvis, and had flooded nearly constantly from that time until she was brought to the hospital, a period of three months. The plan of treatment above described was carried out in her case and, although she was so weak from the loss of blood when the operation was done, that she could remain in the upright position but a few moments at a time, at the end of a month she went out entirely restored to health.

Although it may be "telling tales out of school," I will relate two cases that have resulted fatally during the present winter, by an error in diagnosis on the part of Prof. Braun.

The first was a case of extra-uterine fibroid as large as a child's head, occupying one iliac fossa. In the other iliac fossa extending to the median line, was another tumor of about equal size that fluctuated upon being percussed.

Prof. Braun, in lecturing on this case said : " Gentlemen, we have here evidently to deal with two tumors, one a cyst, and the other solid ; and to further verify our diagnosis it is necessary to ascertain the nature of the contents of this cyst, and with that view I will tap it and draw off a portion of its contents.

Twenty-four hours later, the patient lay a corpse on the table in Prof. Heschl's pathological room. He had tapped a distended and displaced bladder and the patient had rapidly succumbed to peritonitis. The other was a girl, about eighteen years of age, who had never menstrated, though of late she had a slight discharge from the vagina. She complained of great pain in the region of the sacrum, and had latterly suffered considerably in evacuating her bowels. Prof. Braun first attempted a vaginal examination but finding the parts small, and the girl sensitive, introduced his finger into the rectum when he discovered a large tumor, situated as he said, behind the upper walls of the vagina and extending into the cul-de-sac of Douglas. This tumor he concluded was an hæmatocele. To verify this diagnosis he introduced a large sized exploring trochar into it, per rectum.

No fluid making its appearance, he then applied the aspirator to his trochar. Still getting no liquid, he concluded that he was not high enough up, and pushed his instrument still further on. Three days later we had this patient on the table in the dead room.

The post mortem showed that she had a complete atresia of the superior portion of the vagina. The menstrual blood that had escaped from the womb had distended the upper walls of the vagina to an enormous extent. This blood was too thick to pass through the trochar, and when Prof. Braun had pushed his instrument further on, he had punctured the bladder; the urine had infiltrated into the cellular tissue behind the womb, as well as into this artificial sac, setting up a violent cellulitis, followed by a peritonitis, of which the unfortunate girl had died.

Dr. Chiari, who made the post mortems in these cases, very properly remarked that "When so great an authority as Prof. Braun can be so easily mistaken as to the nature of diseases connected with the female generative organs, it certainly behooves men of less ability and experience to be modest in enunciating their opinions, and careful about resorting to any expedient that may prove dangerous to life."

There have been 3,300 women delivered in Prof. B.'s lying-in wards alone during the past year. Of these 29 have died in child-bed. Only one has died of post partum hemorrhage, and one from rupture of the uterus. Nine in all have died of diseases peculiar to the puerperal state; the others dying of diseases that had existed prior to confinement.

The doctor is a thorough believer in the communicability of puerperal fever, of which we have had an outbreak here this winter. During its prevalence all students were excluded from the examination of pregnant women, the lying-in wards were abandoned, thoroughly cleansed and re-calcimined. In three weeks the disease was eliminated from the hospital.

The careful post mortem examinations made here, on all women who die of acute puerperal processes, whether of an epidemic or sporadic character, whether eventuating in peritonitis, uterine phlebitis, or general septicæmia, seem to show that these affections take their origin in inflammatory and septic changes within the body of the womb itself. In view of this fact, both Professors Braun and Spaeth treat all these cases by a thorough drainage and cleansing of the intra-uterine surface.

Whenever the discharges in a puerperal case, by their odor or otherwise, indicate that they are becoming septic in character, the womb is thoroughly washed out with a 2 per cent. solution of carbolic acid.

The injections are made through a No. 12 English male elastic catheter, and this catheter is left constantly in the womb to act as a drainage tube, being removed, well cleansed and the injection repeated once in from eight to twelve hours.

W. S. Caldwell.

---

According to a statement in the Academy two healthy men allowed their limbs to be coated with impermeable plasters while the trunk was varnished with several layers of flexible collodium, which were allowed to remain for a week. None of the evil consequences observed in animals, made their appearance; there was no fall of temperature, no albuminuria, no exhaustion, no dyspnœa, convulsion or paralysis.

The doctors of Havre have united and issued a circular to their patients threatening a general "strike" unless their terms are complied with. From $2.00 to $4.00 for night and urgent visits is the price demanded

Dr. James Nevins Hyde will hold a special clinique for cutaneous and venereal diseases at Rush Medical College, every Monday throughout the year, at 2½ p. m., commencing with the 1st inst.

Dr. R. L. Rea has been recently appointed Professor of Anatomy in the Chicago Medical College.

